# Supplementary material for: Deep-sea gas hydrate mounds and chemosynthetic fauna discovered at 3640 m on the Molloy Ridge, Greenland Sea
Source: Nat Commun. 2025 Dec 17;16:11287. doi: 10.1038/s41467-025-67165-x (PMC12722328; doi:10.1038/s41467-025-67165-x)
Supplement: Supplementary file 1 — Supplementary Information [file 41467_2025_67165_MOESM1_ESM.pdf]

Supplementary Information to:

Deep-sea gas hydrate mounds and chemosynthetic fauna discovered at 3640 m on the Molloy Ridge, Greenland Sea

Giuliana Panieri[1,21], Jonathan T. Copley[2], Katrin Linse[3], Verity Nye[4], Eva Ramirez-Llodra[5], Claudio Argentino[1], Bénédicte Ferré[1], the Arctic Deep - Extreme24 consortium* and Alex D. Rogers[5,22]

Corresponding authors: Giuliana Panieri (giuliana.panieri@uit.no) and Jonathan T. Copley (jtc@southampton.ac.uk)

Affiliations

[1]Department of Geosciences, UiT The Arctic University of Norway, Tromsø, Norway

[2]School of Ocean & Earth Science, University of Southampton, Southampton, United Kingdom

[3]British Antarctic Survey, Cambridge, UK

[4]Ocean Census, Begbroke Science Park, Oxfordshire, UK

[5]REV Ocean, Fornebu, Norway

[21]CNR, ISP Institute of Polar Science, Campus Scientifico-Università Ca' Foscari Venezia, Mestre, Italy (present address)

[22]National Oceanography Centre Southampton UK (present address)

*A list of authors and their affiliations appears at the end of the Supplementary Information

Supplementary Figures

Supplementary Figure 1. ROV frame showing the gas hydrate sampling. a ROV frame showing the sampling event of the gas hydrate from the flank of one of the Freya gas hydrate mounds with a blade corer manipulated by the ROV arm. The coordinates on the ROV frame indicate where the sample has been taken. **b** The image shows the successful retrieval of the gas hydrate sample using the blade corer (**a**) once on deck The photo has been taken by Alex D. Rogers. The sample has been used for the oil and gas analyses presented in this paper, as reported in Supplementary Figures 2-3 and Figure 4.

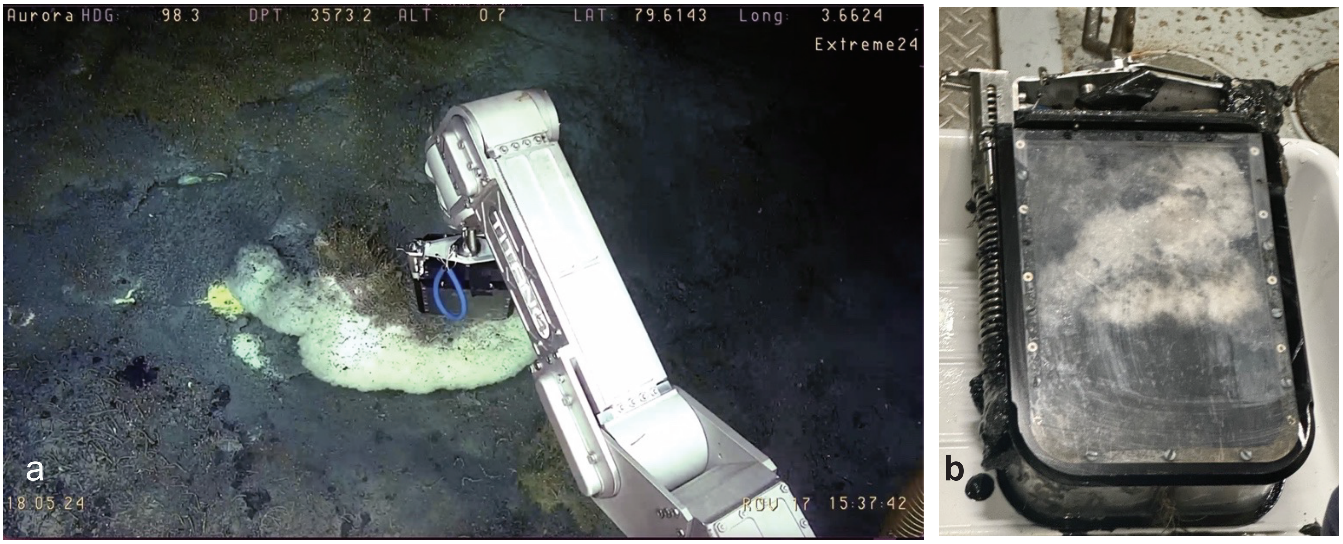

Supplementary Figure 2. **n-Alkanes chromatograms of the oil from Freya gas hydrate mounds**. Extract gas chromatograms showing n-alkanes distribution in oil extracted from **a** hydrate sample and **b** in the sediment (both collected from blade cores). The oil distribution shows a gas condensate signal with unusually abundant acyclic isoprenoids. The sediment shows odd-over-even preference among n-C_{23}-n-C_{33} members, consistent with a contribution from immature higher plant material.

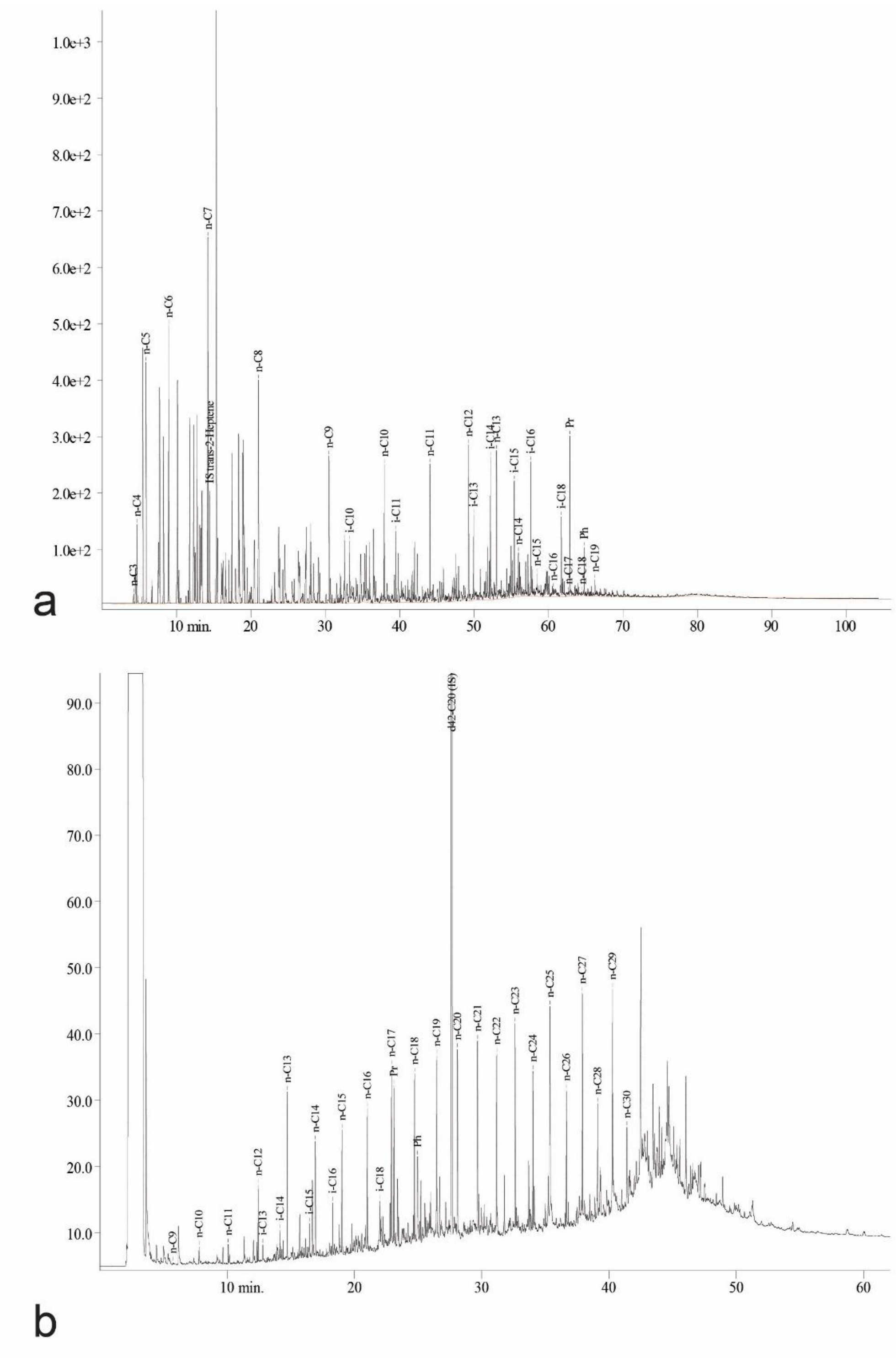

Supplementary Figure 3. Source rock proxies. a Sterane ternary diagram of the percentage of C_{27}, C_{28}, and C_{29} obtained from the m/z 218 fragmentogram used to assess the paleo-depositional environment of the source rock[1]. The steranes of Molløy seep point to a transitional marine-terrestrial environment whereas the sediment sample displays a more open marine composition. b) Tetracyclic polyprenoid (TPP) ratio (TPP = 2(TPPa)/(2(TPPa) + 27-norC_{26}) versus content of C_{30} 24-n-propyldiacholestanes (C_{30} /(C_{27} + C_{28} + C_{29} + C_{30}) for oil samples indicating an evident fresh/brackish association (correlating with abundant angiosperm inputs) with some minor marine contribution. c) C_{31}/C_{30} hopanes versus C_{26}/C_{25} tricyclic terpanes (cheilanthanes) plot showing a distinct lacustrine contribution.

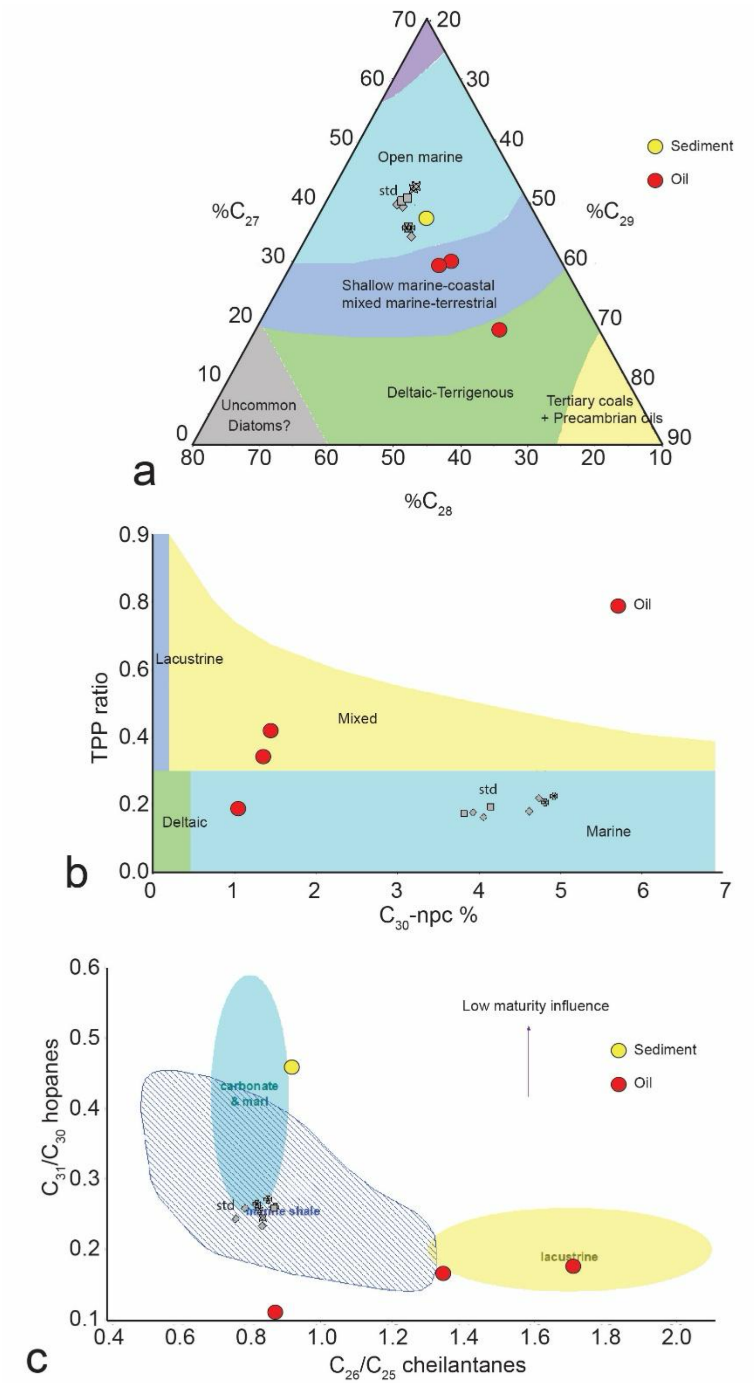

Supplementary Figure 4. **Oil maturity proxies**. Maturity indications from isomerism of hopanes (a) and steranes (b) suggesting a wet-gas/pre-oil window.

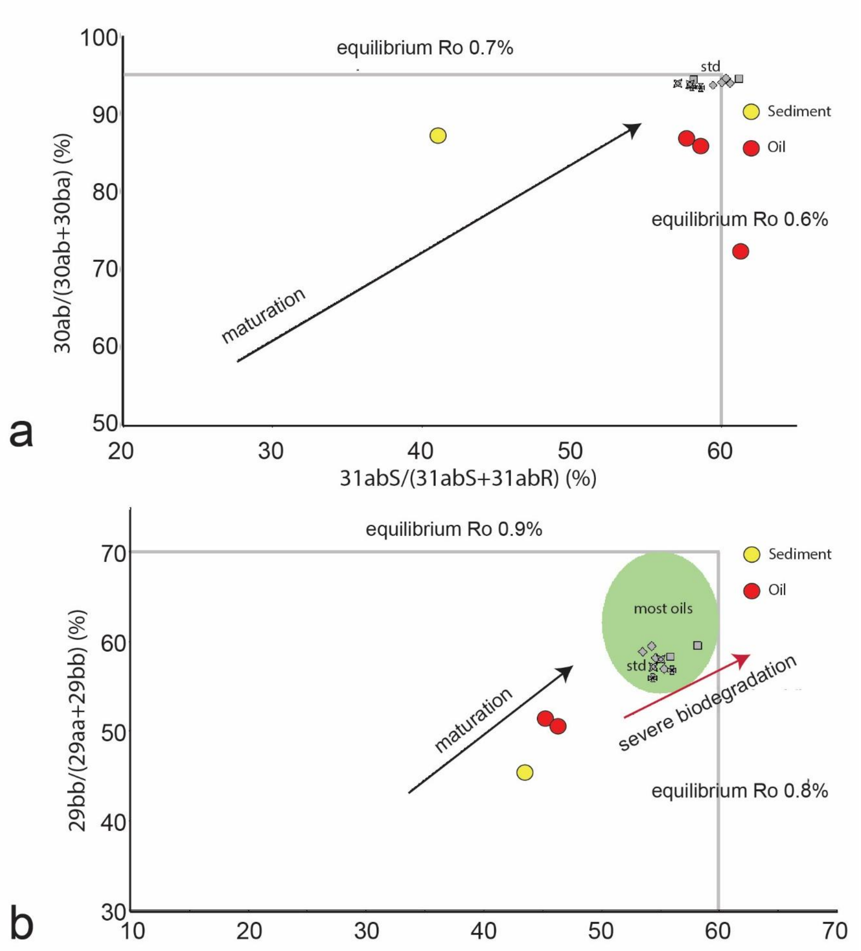

Supplementary Figure 5. All-pairwise faunal similarity matrix (Sørensen Index values) for high-Arctic (>72 °N) cold seeps and hydrothermal vents, calculated from family presence/absence data using faunal records from this study and published literature (see Table 2 for data sources). Darker shading indicates higher % similarity values. Freya = Freya gas hydrate mounds; Jøtul = Jøtul vent field; Vestnesa = Vestnesa Ridge seeps; PKF = Prins Karls Forland seeps; S+B = Storfjordrenna+Bjørnøyrenna (data combined for analysis as separate inventories are unavailable in the literature); HMMV = Håkon Mosby Mud Volcano; Loki = Loki's Castle vent field; Aurora = Aurora Vent Field

	Freya	*Jøtul*	*Vestnesa*	*PKF*	*S+B*	*HMMV*	*Loki*
Jøtul	59						
Vestnesa	46	29					
PKF	17	11	11				
S+B	26	12	38	20			
HMMV	29	18	32	11	32		
Loki	47	32	27	23	29	41	
Aurora	29	38	13	0	0	4	19

Supplementary Figure 6. Origin of the flare at Freya gas hydrate mounds. Fledermaus plot showing where the flares originate on the topography as processed with Qimera and acoustic backscatter processed with FMMidwater using the shipboard MBES at the Freya gas hydrate mounds (79.6º N, depth 3640 m).

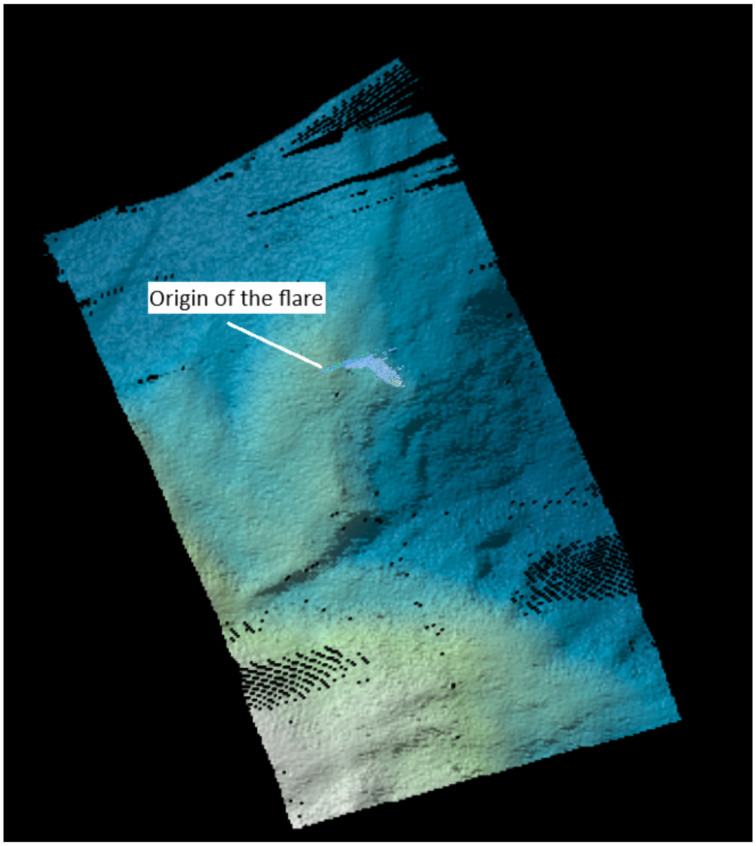

References

1. Huang, W.-Y. & Meinschein, W. G. Sterols as ecological indicators. *Geochimica et Cosmochimica Acta* **43**, 739–745 (1979).

Names and affiliations of Arctic Deep – EXTREME 24 consortium

Alejandra Saenz de Tejada[6], Alex David Rogers[4,22], Alfredo Rosales Ruiz[7], Asgeir Steinsland[8], Carlotta Redaelli[1,9], Clarisse Goar[10], Daniel Despujois[6], Eva Ramirez-Llodra[5], Ewan McEvoy[11], Fereshteh Hemmateenejad[9], Giuliana Panieri[1,21], Ida Søhol[12], Ines Barranchea Angeles[1], Jack Hogan[13], Jessica Michelle Webster[14], Joe Sharman[15], Jonathan T. Copley[3], Katrin Linse[4], Laura Warmuth[16], Lawrence Hislop[5], Leif Johan Ohnstad[8], Leighton Rolley[5], Martin Hartley[15], Nuria Rico Seijo[13], Pamela Rivadeneira[17], Patricia Esquete Garrote[18], Patrick Vågenes[5], Pedro Furtado Costa Rodrigues[14], Raissa Hogan[19], Stig Vågenes[5], Tor-Arve Lunde[5], Usha Parameswaran[20], Verity Nye[15], Will West[15]

[1]Department of Geosciences UiT The Arctic University of Norway Tromsø Norway

[2]School of Ocean & Earth SciencesSchool of Ocean & Earth ScienceUniversity of Southampton Southampton UK

[3]British Antarctic Survey Cambridge UK

[4]Ocean Census Begbroke Science Park Oxfordshire UK

[5]REV Ocean Fornebu Norway

[6]CIIMAR Terminal de Cruzeiros do Porto de Leixões Matosinhos Porto Portugal

[7]Fundación Museo del Mar de Ceuta Ceuta Spain

[8]Institute of Marine Research Bergen Norway

[9]Department of Earth and Environmental Sciences (DISAT)University of Milano Bicocca Milan Italy

[10]IFREMER Plouzané France

[11]School of Biological and Environmental Sciences Liverpool John Moores University Liverpool UK

[12]Department of Arctic and Marine Biology The Arctic University of Norway Tromsø Norway

[13]Nekton Begbroke Science Park Oxfordshire UK

[14]BBC Natural History Film Unit Bristol UK

[15]The Nippon Foundation-Nekton Ocean Census Programme Begbroke Science Park Oxfordshire UK

[16]Department of Biology University of Oxford Oxford UK

[17]Laboratorio de Ecosistemas Costeros, Plataforma y Mar profundo Museo Argentino de Ciencias Naturales “Bernardino Rivadavia” (CONICET) Buenos Aires Argentina

[18]Departamento de Biologia & CESAM (Centro de estudos do Ambiente e do Mar) Universidade de Aveiro Aveiro Portugal

[19]School of Natural Sciences University of Galway Galway Ireland

[20]Centre for Polar Ocean Research (NCPOR), Ministry of Earth Sciences Government of India Vasco-da-Gama GoaI ndia

[21]CNR, ISP Institute of Polar Science Campus Scientifico-Università Ca’ Foscari Venezia Mestre Italy

[22]National Oceanography Centre Southampton UK
