# Supplementary material for: Deep-sea gas hydrate mounds and chemosynthetic fauna discovered at 3640 m on the Molloy Ridge, Greenland Sea
Source: Nat Commun. 2025 Dec 17;16:11287. doi: 10.1038/s41467-025-67165-x (PMC12722328; doi:10.1038/s41467-025-67165-x)
Supplement: Supplementary file 2 — Description of Additional Supplementary Files [file 41467_2025_67165_MOESM2_ESM.pdf]

Description of Additional Supplementary Files

File Name: Supplementary Data 1
Description: Faunal families recorded at high-Arctic (>72 °N) seeps (s) and vents (v). Inventories for Freya and Jøtul from this study; data for other sites compiled from published papers (Vestnesa Ridge [16] [66]; Prins Karls Forland [66] [8] [36]; Storfjordrenna+Bjørnøyrenna [8] [66] [67]; Håkon Mosby Mud Volcano [68]; Loki's Castle [22]; Aurora Vent Field [21]). Data for Storfjordrenna and Bjørnøyrenna are combined because separate inventories are unavailable in the literature. Taxa not identified to family level are excluded to avoid conflation in family-level similarity analysis.

File Name: Supplementary Movie 1
Description: **Trains of methane bubbles emitted from Freya gas hydrate mounds.** A video of methane bubbles emitted from Freya gas hydrate mounds was recorded while samples were collected for biological investigations. The video was recorded by the SubVIS Orca, IP Zoom HD Camera mounted on the ROV.
