# Supplementary material for: Deep-sea gas hydrate mounds and chemosynthetic fauna discovered at 3640 m on the Molloy Ridge, Greenland Sea
Source: Nat Commun. 2025 Dec 17;16:11287. doi: 10.1038/s41467-025-67165-x (PMC12722328; doi:10.1038/s41467-025-67165-x)
Supplement: Supplementary file 5 — Reporting Summary [file 41467_2025_67165_MOESM5_ESM.pdf]

nature portfolio

Corresponding author(s): NCOMMS-24-65734B
Last updated by author(s): October 14, 2025

Reporting Summary

Nature Portfolio wishes to improve the reproducibility of the work that we publish. This form provides structure for consistency and transparency in reporting. For further information on Nature Portfolio policies, see our Editorial Policies and the Editorial Policy Checklist.

Statistics

For all statistical analyses, confirm that the following items are present in the figure legend, table legend, main text, or Methods section.

n/a	Confirmed	
☐	☒	The exact sample size (n) for each experimental group/condition, given as a discrete number and unit of measurement
☒	☐	A statement on whether measurements were taken from distinct samples or whether the same sample was measured repeatedly
☐	☒	The statistical test(s) used AND whether they are one- or two-sided<br>*Only common tests should be described solely by name; describe more complex techniques in the Methods section.*
☒	☐	A description of all covariates tested
☒	☐	A description of any assumptions or corrections, such as tests of normality and adjustment for multiple comparisons
☒	☐	A full description of the statistical parameters including central tendency (e.g. means) or other basic estimates (e.g. regression coefficient) AND variation (e.g. standard deviation) or associated estimates of uncertainty (e.g. confidence intervals)
☐	☒	For null hypothesis testing, the test statistic (e.g. F, t, r) with confidence intervals, effect sizes, degrees of freedom and P value noted<br>*Give P values as exact values whenever suitable.*
☒	☐	For Bayesian analysis, information on the choice of priors and Markov chain Monte Carlo settings
☒	☐	For hierarchical and complex designs, identification of the appropriate level for tests and full reporting of outcomes
☐	☒	Estimates of effect sizes (e.g. Cohen's d, Pearson's r), indicating how they were calculated

Our web collection on statistics for biologists contains articles on many of the points above.

Software and code

Policy information about availability of computer code

Data collection	*Provide a description of all commercial, open source and custom code used to collect the data in this study, specifying the version used OR state that no software was used.*
Data analysis	PRIMER Version 7 for multivariate analysis

For manuscripts utilizing custom algorithms or software that are central to the research but not yet described in published literature, software must be made available to editors and reviewers. We strongly encourage code deposition in a community repository (e.g. GitHub). See the Nature Portfolio guidelines for submitting code & software for further information.

Data

Policy information about availability of data

All manuscripts must include a data availability statement. This statement should provide the following information, where applicable:

- Accession codes, unique identifiers, or web links for publicly available datasets
- A description of any restrictions on data availability
- For clinical datasets or third party data, please ensure that the statement adheres to our policy

Provide your data availability statement here.

Research involving human participants, their data, or biological material

Policy information about studies with human participants or human data. See also policy information about sex, gender (identity/presentation), and sexual orientation and race, ethnicity and racism.

Reporting on sex and gender	*Use the terms sex (biological attribute) and gender (shaped by social and cultural circumstances) carefully in order to avoid confusing both terms. Indicate if findings apply to only one sex or gender; describe whether sex and gender were considered in study design; whether sex and/or gender was determined based on self-reporting or assigned and methods used. Provide in the source data disaggregated sex and gender data, where this information has been collected, and if consent has been obtained for sharing of individual-level data; provide overall numbers in this Reporting Summary. Please state if this information has not been collected. Report sex- and gender-based analyses where performed, justify reasons for lack of sex- and gender-based analysis.*
Reporting on race, ethnicity, or other socially relevant groupings	*Please specify the socially constructed or socially relevant categorization variable(s) used in your manuscript and explain why they were used. Please note that such variables should not be used as proxies for other socially constructed/relevant variables (for example, race or ethnicity should not be used as a proxy for socioeconomic status). Provide clear definitions of the relevant terms used, how they were provided (by the participants/respondents, the researchers, or third parties), and the method(s) used to classify people into the different categories (e.g. self-report, census or administrative data, social media data, etc.) Please provide details about how you controlled for confounding variables in your analyses.*
Population characteristics	*Describe the covariate-relevant population characteristics of the human research participants (e.g. age, genotypic information, past and current diagnosis and treatment categories). If you filled out the behavioural & social sciences study design questions and have nothing to add here, write "See above."*
Recruitment	*Describe how participants were recruited. Outline any potential self-selection bias or other biases that may be present and how these are likely to impact results.*
Ethics oversight	*Identify the organization(s) that approved the study protocol.*

Note that full information on the approval of the study protocol must also be provided in the manuscript.

Field-specific reporting

Please select the one below that is the best fit for your research. If you are not sure, read the appropriate sections before making your selection.

☐ Life sciences ☐ Behavioural & social sciences ☒ Ecological, evolutionary & environmental sciences

For a reference copy of the document with all sections, see nature.com/documents/nr-reporting-summary-flat.pdf

Ecological, evolutionary & environmental sciences study design

All studies must disclose on these points even when the disclosure is negative.

Study description	This study documents the discovery of the world's deepest known seafloor hydrate mounds at 3640 m on the Mølloy Ridge and their associated chemosynthetic fauna. It provides new insights into the geological and ecological dynamics of deep-sea methane hydrate systems, revealing that these Arctic seep communities share greater taxonomic similarity with hydrothermal vent fauna than with shallower methane seeps.
Research sample	The research sample in this study includes both geological and biological specimens collected from the deep-sea hydrate mounds on the Mølloy Ridge at a depth of 3640 m. The geological samples comprise methane hydrate deposits, gas bubbles, and crude oil, which were analyzed for isotopic composition to determine the source and characteristics of the methane. The biological samples include a diverse assemblage of cold-seep fauna, such as siboglinid and maldanid tubeworms, skeneid and rissoid snails, and melitid amphipods, which were identified and compared with known Arctic hydrothermal vent and seep communities. Additional biological samples were collected from the Jøtul vent field on the Knipovich Ridge for comparative analysis.
Sampling strategy	The sampling strategy comprises a combination of in situ observations, targeted geological and biological sampling.
Data collection	Data for this study were collected aboard the RV Kronprins Haakon using sonar mapping, hydrographic profiling, and ROV-based sampling at the Freya gas hydrate mounds and Jøtul vent field. Specifically, we did: seafloor mspping using A Kongsberg EM302 multibeam echosounder identified gas flares and mapped the seafloor; the ydrographic profiling was done with Seabird 911 Plus CTD probe that recorded temperature and salinity in the water column. The ROV Sampling was done with the Aurora ROV that captured high-resolution video and collected biological, gas hydrate, sediment, and oil samples using pushcores, a blade corer, suction samplers, and a manipulator arm. The sample processing for faunal specimens identification started with preservation for taxonomic analysis, while gas, sediment, and oil samples were analyzed for chemical composition and isotopic signatures. Several operators were involved in the different steps of data collections.
Timing and spatial scale	Data collection took place in May 2024, with sampling at the Jøtul vent field on May 12–13 and at the Freya gas hydrate mounds on May 18. Sampling operations were conducted whenever we were onsite, utilizing the 12-hour operation of the ROV, while seafloor mapping and CTD profiling were performed during the night. Regarding the spatial scale, we took all the samples we could from the

	gas hydrate mounds, coverign an area of ~100 x 100 m2 at a seafloor depth from 3570 to 3747 m.
Data exclusions	We did not exclude data.
Reproducibility	All operations and sample collection methods in this study are fully reproducible. All the data collected (seafloor mapping, gas flare detection, and hydrographic profiling) and samples taken followed standard protocols to ensure consistent and reproducible sample acquisition.
Randomization	The sample collection was designed to minimize bias and ensure a representative assessment of the Freya gas hydrate mounds and Jøtul vent field. Although the sampling locations were chosen based on the identified presence of gas hydrate mounds and gas seeps, sample acquisition was randomized to account for potential variability in biological and geochemical features.
Blinding	Blinding was not explicitly applied during the field sampling or analysis phases. Given the nature of the research, where sample collection and subsequent analysis were largely observational, blinding was not necessary for data collection or sample identification

Did the study involve field work? ☒ Yes ☐ No

Field work, collection and transport

Field conditions	The fieldwork for this study was conducted under challenging Arctic deep-sea conditions aboard the RV Kronprins Haakon. The operations took place in remote regions of the Arctic, at depths of 3640 m at the Mølloy Ridge and the Jøtul vent field.<br>The weather conditions during the expedition were variable, with cold temperatures, sea ice, and rough seas typical of the Arctic environment. Despite these challenges, the Kronprins Haakon provided a stable platform for operations, and the ROV Aurora was successfully deployed from the ship's moonpool, which allowed for operations in sea ice and adverse weather conditions. The ROV was able to operate for up to 12 hours per dive, making it possible to conduct extended surveys and sample collection during each deployment. Overall, the field conditions were demanding and required skilled operators.
Location	Freya Gas Hydrate Mounds are located at approximately 79.6930°N, 3.6617°E on the Mølloy Ridge, at a depth of 3640 meters.
Access & import/export	Accessing the Freya gas hydrate mounds and the Jøtul vent field required significant logistical planning and effort, given the remote Arctic location and deep-sea environments. The sampling was conducted aboard the RV Kronprins Haakon using shipr instruments and the ROV Aurora from REV Ocean. The samples were preserved and processed according to established protocols indicated in the methods section of the manuscript, and all specimens and geochemical samples were left in Tromsø, Norway, at the end of the cruise where UiT, the University of Norway, served as the central location for further analysis. This process was in accordance with the agreement with Ocean Census, ensuring that all specimens and samples remained in Tromsø for taxonomic and ecological analysis.
Disturbance	To minimize the risk of disturbance during sampling operations the ROV Aurora was used for targeted sampling, which ensured minimal disturbance to the seabed.

Reporting for specific materials, systems and methods

We require information from authors about some types of materials, experimental systems and methods used in many studies. Here, indicate whether each material, system or method listed is relevant to your study. If you are not sure if a list item applies to your research, read the appropriate section before selecting a response.

Materials & experimental systems

n/a	Involved in the study	
☒	☐	Antibodies
☒	☐	Eukaryotic cell lines
☒	☐	Palaeontology and archaeology
☒	☐	Animals and other organisms
☒	☐	Clinical data
☒	☐	Dual use research of concern
☒	☐	Plants

Methods

n/a	Involved in the study	
☒	☐	ChIP-seq
☒	☐	Flow cytometry
☒	☐	MRI-based neuroimaging

Plants

Seed stocks

Report on the source of all seed stocks or other plant material used. If applicable, state the seed stock centre and catalogue number. If plant specimens were collected from the field, describe the collection location, date and sampling procedures.

Novel plant genotypes

Describe the methods by which all novel plant genotypes were produced. This includes those generated by transgenic approaches, gene editing, chemical/radiation-based mutagenesis and hybridization. For transgenic lines, describe the transformation method, the number of independent lines analyzed and the generation upon which experiments were performed. For gene-edited lines, describe the editor used, the endogenous sequence targeted for editing, the targeting guide RNA sequence (if applicable) and how the editor was applied.

Authentication

Describe any authentication procedures for each seed stock used or novel genotype generated. Describe any experiments used to assess the effect of a mutation and, where applicable, how potential secondary effects (e.g. second site T-DNA insertions, mosiacism, off-target gene editing) were examined.
